# The role of contactin-associated protein-like 2 in neurodevelopmental disease and human cerebral cortex evolution

**DOI:** 10.3389/fnmol.2022.1017144

**Published:** 2022-10-20

**Authors:** Frances St. George-Hyslop, Toomas Kivisild, Frederick J. Livesey

**Affiliations:** ^1^Zayed Centre for Research Into Rare Disease in Children, UCL Great Ormond Street Institute of Child Health, University College London, London, United Kingdom; ^2^Temerty Faculty of Medicine, University of Toronto, Toronto, ON, Canada; ^3^Estonian Biocentre, Institute of Genomics, University of Tartu, Tartu, Estonia; ^4^Department of Human Genetics, KU Leuven, Leuven, Belgium

**Keywords:** autism (ASD), neurodevelopmental disorders, cerebral cortex, genetics, *CNTNAP2*

## Abstract

The contactin-associated protein-like 2 *(CNTNAP2)* gene is associated with multiple neurodevelopmental disorders, including autism spectrum disorder (ASD), intellectual disability (ID), and specific language impairment (SLI). Experimental work has shown that *CNTNAP2* is important for neuronal development and synapse formation. There is also accumulating evidence for the differential use of *CNTNAP2* in the human cerebral cortex compared with other primates. Here, we review the current literature on *CNTNAP2*, including what is known about its expression, disease associations, and molecular/cellular functions. We also review the evidence for its role in human brain evolution, such as the presence of eight human accelerated regions (HARs) within the introns of the gene. While progress has been made in understanding the function(s) of *CNTNAP2*, more work is needed to clarify the precise mechanisms through which *CNTNAP2* acts. Such information will be crucial for developing effective treatments for *CNTNAP2* patients. It may also shed light on the longstanding question of what makes us human.

## Introduction

**FIGURE 1:**
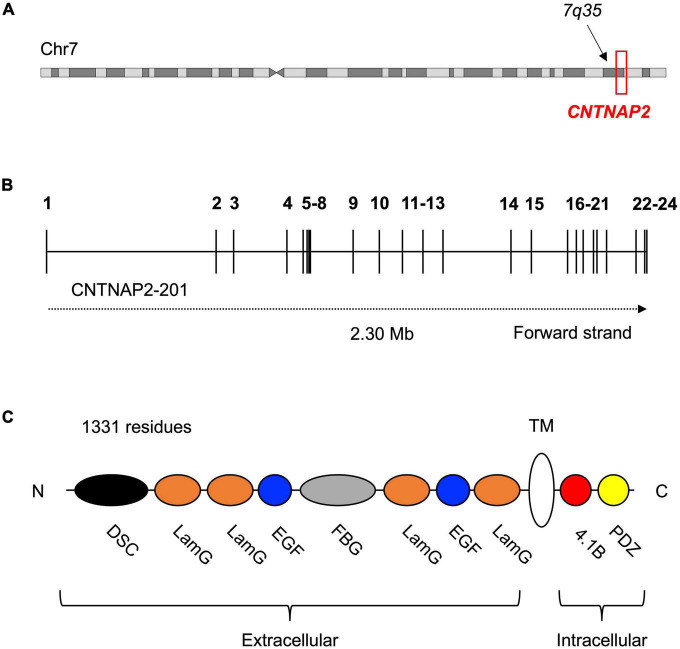
Location and structure of the contactin-associated protein-like 2 (*CNTNAP2*) gene and protein. **(A)** Contactin-associated protein-like 2 is located at the distal end of the long arm (q) of chromosome 7 (GRCh38 chr7: 146,116,002–148,420,998). **(B)** Schematic of the 24 exons (numbered) and 23 introns of the *CNTNAP2* gene. Transcript CNTNAP2-201 is the canonical transcript. **(C)** Structure of the contactin-associated protein-like 2 (CASPR2) protein and its eight extracellular, one transmembrane, and two intracellular subdomains. The extracellular subdomains include a discoidin domain (DSC) and a fibrinogen-like domain (FBG), both of which are known to facilitate cell-cell adhesion and interactions with the extracellular matrix. The remaining extracellular subdomains are four laminin-G domains (LamG) and two epidermal growth factor-like domains (EGF). These are predicted to mediate receptor-ligand interactions and cell adhesion, migration, and differentiation. The intracellular region of CASPR2 is mostly involved in protein-protein interactions, as it contains a type II PDZ domain (PDZ) and a protein 4.1B binding site (4.1B). N-terminus (N); C-terminus (C).

The contactin-associated protein-like 2 gene *(CNTNAP2)* is located at chromosome 7q35. *CNTNAP2* spans 2.3 Mb across 24 exons and is the one of the largest genes in the genome (Figures 1A,B; Rodenas-Cuadrado et al., 2014). Mutations in *CNTNAP2* have been linked to neurodevelopmental disorders like autism spectrum disorder (ASD), intellectual disability (ID), and specific language impairment (SLI). There is also growing evidence that the temporal and spatial expression of *CNTNAP2* during brain development in humans is different from other primates. The potential evolutionary significance of *CNTNAP2* is further highlighted by the presence of eight human accelerated regions (HARs) in its introns. HARs are DNA sequences that are highly conserved across primates or mammals, but have an unexpectedly large number of human-specific nucleotide changes (see section “*What are HARs?*” for a full description) (Pollard et al., 2006; Prabhakar et al., 2006; Bird et al., 2007; Bush and Lahn, 2008; Lindblad-Toh et al., 2011). Given the association between *CNTNAP2* and human cognitive disorders, and the evidence for its potential role in human evolution, this review aims to synthesize clinical, experimental, and evolutionary data to understand *CNTNAP2* function and the biological consequences of its disruption.

### The contactin-associated protein-like 2 protein

Contactin-associated protein-like 2 encodes the contactin-associated protein-like 2 (CASPR2) protein. CASPR2 is a single-pass transmembrane protein composed of 1,331 residues and with a mass of 138 kDa (Rodenas-Cuadrado et al., 2014). The protein contains eight extracellular, one transmembrane, and two intracellular subdomains (Figure 1C). CASPR2 belongs to the neurexin superfamily of transmembrane proteins, which are cell adhesion molecules involved in synapse formation and function. Several other CASPR proteins exist (CASPR1 – 5), however, these proteins have fewer disease associations than CASPR2 and there is less evidence for their involvement in human evolution. They also all share less than 50% sequence identity to CASPR2, implying that CASPR2 serves differing functions.

CASPR2 is highly conserved amongst mammals – for example, human and mouse amino acid sequences are 94% identical (Rodenas-Cuadrado et al., 2014). This conservation is even greater between humans and chimpanzees, with only 6/1,331 residues differing (99.5% identity). Comparisons with archaic humans show yet further conservation. Between both Denisovans and Neanderthals, only one amino acid difference is noted with modern humans. Residue 345 is a valine in *Homo sapiens* but isoleucine in the two other species. One other noteworthy difference exists: position 215 is an asparagine in humans and Denisovans, but in all other species (including Neanderthals) it is a Lysine. It is currently unclear whether either of these amino acid changes have had functional consequences. However, PolyPhen-2 predicts both 345-I and 215-N to be functionally neutral (Adzhubei et al., 2010).

Although the molecular function(s) of CASPR2 are not completely understood, it was first characterized in the axon initial segment (AIS) and juxtaparanodal regions of myelinated neurons in the peripheral nervous system (Poliak et al., 1999). At juxtaparanodes, CASPR2 forms a complex with the contactin-2 protein (CNTN2). CASPR2 also binds to contactin-1 (CNTN1), an interaction which may play a role in nerve myelination, but this is currently not well characterized (Rubio-Marrero et al., 2016). Conversely, the CASPR2-CNTN2 complex has been robustly demonstrated to be required for the clustering of voltage-gated K^+^ channels, which function in the conduction of nerve impulses (Strauss et al., 2006). Several studies have reported that *Cntnap2* knockout mice have a significant reduction in the density of Kv1.2 potassium channels in cortical myelinated axons (Poliak et al., 2003; Scott et al., 2019). Scott et al. (2019) observed these changes correlated with an increase in excitatory transmission and increased probability of neurotransmitter release. However, given that *CNTNAP2* expression is high in development - at timepoints prior to nerve myelination - other developmental functions are also likely to exist. These include proposed roles in neurite outgrowth and in the formation of synaptic connections (both discussed later in this review).

Lastly, recent work has identified that synaptic potentiation triggers the N-terminal region of CASPR2 to be cleaved (Martín-de-Saavedra et al., 2022). This ecto-domain localizes extracellularly near synapses, and binds and activates the PMCA calcium extrusion pump. In other words, synaptic activity causes CASPR2 to promote calcium export from neurons, thereby suppressing excitability and network activity. A loss of CASPR2 could therefore lead to hyperexcitability by increasing intracellular calcium. This finding is especially noteworthy in light of the neuronal over-excitation frequently reported in the brains of autistic individuals (Rodenas-Cuadrado et al., 2016), and could relate to the seizures that commonly occur in individuals with *CNTNAP2* loss-of-function mutations. Accordingly, the levels of the cleaved CASPR2 N-terminus are reduced in the cerebral spinal fluid of individuals with ASD, which further supports this hypothesis (Martín-de-Saavedra et al., 2022).

Other proteins that interact with CASPR2 include the scaffolding protein PAR3 (Gao et al., 2020) and the serine protein kinase CASK (Gao et al., 2019). These interactions may be required for the correct localization of CASPR2 within developing neurons – either intracellularly or on the plasma membrane, respectively – as mis-localization of CASPR2 occurs with a loss of their binding (Gao et al., 2019, 2020). Immunoprecipitation experiments have also suggested CASPR2 binds intracellularly to ADAM22, LGI1, GPR37, and subunits of the Kv1.1 channel (KCNA1) (Poot, 2017). Extracellularly, CASPR2 binds to the scaffolding proteins DLG1 and DLG4. However, the precise functions of these interactions are not currently known.

### Contactin-associated protein-like 2 expression in the human, primate, and rodent cortex

Although *CNTNAP2* was first described in the peripheral nervous system (Poliak et al., 1999), it is primarily expressed in the cerebral cortex (Bakkaloglu et al., 2008; Vernes et al., 2008). In adult humans, the highest expression is observed in layers II–V of the frontal and temporal cortices. Sub-cortically, *CNTNAP2* is also present in the thalamus, amygdala, and striatum. Interestingly, this pattern of expression is dramatically restricted to the cortico-striato-thalamic circuitry that mediates higher cognitive functions (Abrahams et al., 2007).

In the human fetal brain, *CNTNAP2* is expressed in the frontal and anterior temporal lobes, medial ganglionic eminence, striatum, and dorsal thalamus (Alarcon et al., 2008). This anterior cortical enrichment is not observed in rodents. In the developing mouse and rat cortex, *Cntnap2* is broadly expressed throughout the brain and is low or absent in the cortical plate (with highest expression – when present – located posteriorly) (Abrahams et al., 2007). Even in adulthood, *Cntnap2* is never enriched in the rodent frontal cortex, unlike in humans.

In addition to the differences in *CNTNAP2* expression between humans and rodents, differential expression has also been observed between humans and non-human primates. Recent single cell RNA-Seq (scRNA-Seq) data revealed *CNTNAP2* is significantly increased in the excitatory neurons of 2 month old cortical organoids derived from humans versus chimpanzees (Kanton et al., 2019). Increased expression in human organoids was also identified relative to macaque for both excitatory neurons and interneurons. *CNTNAP2* expression was also significantly higher in chimpanzee excitatory neurons than in macaque. These findings suggest that cortical *CNTNAP2* expression has increased along the lineage leading to humans.

Due to the limits on accessing primate fetal cortex, only one scRNA-Seq dataset of primary human and non-human primate developing brain currently exists (Pollen et al., 2019). This study includes matched samples from macaque (post-conception weeks 8–24) and human (post-conception weeks 4–40). In both deep and upper layer excitatory neurons, *CNTNAP2* is increased in human relative to macaque. However, neither remain significant after correction for multiple testing. As additional transcriptomic studies of human and non-human primate fetal brain become available, this relationship can be tested further.

In primary adult cortex, scRNA-Seq of human, chimpanzee, and macaque does not show evidence of increased expression in humans. Indeed, in a study by Kanton et al. (2019) the only significant differences identified were decreases in human *CNTNAP2* expression. In chimpanzees, excitatory layer 6a neurons showed significantly increased *CNTNAP2* compared to human. In macaques, excitatory layers 1, 3e, and 6b as well as inhibitory layers 4b and 6b all had increased expression relative to human. The lack of increased human *CNTNAP2* expression in adulthood reinforces that a human-specific role for *CNTNAP2* is most likely occurring during cortical development.

It is important to note, however, that *CNTNAP2* has multiple protein-coding isoforms that cluster at the 3’ end of the locus. As most scRNA-Seq technology only detects the 3’ end of a transcript, the different isoforms cannot be distinguished. Thus, the above scRNA-seq expression data of *CNTNAP2* should be interpreted with this in mind.

### Contactin-associated protein-like 2 mutations are linked to human neurodevelopmental disorders

Individuals with *CNTNAP2* loss-of-function mutations typically display four core phenotypes: (1) ID (Lu et al., 2021), (2) ASD (O’Roak et al., 2011), (3) SLI (Centanni et al., 2015), and (4) epilepsy (Friedman et al., 2008). Other disorders associated with *CNTNAP2* include schizophrenia (Lee et al., 2015), attention deficit hyperactivity disorder (ADHD) (Elia et al., 2010), Tourette syndrome (Verkerk et al., 2003), dyslexia (Veerappa et al., 2013), and major depression (Rodenas-Cuadrado et al., 2014). These illnesses have been identified in individuals with microdeletions or point mutations affecting only the *CNTNAP2* locus.

**FIGURE 2:**
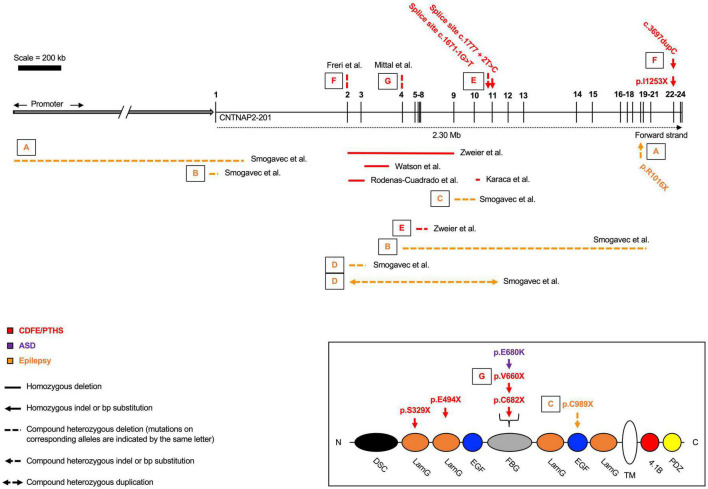
Homozygous and compound heterozygous contactin-associated protein-like 2 (*CNTNAP2*) mutations. Mutations affecting the *CNTNAP2* locus are shown with their associated disorders. Mutations are commonly found in the middle of the gene, between exons 2 – 13. Cortical dysplasia focal epilepsy/Pitt-Hopkins syndrome (CDFE/PTHS) are the most common diagnoses, though patients typically fall into multiple categories. Mutations that map to a subdomain of the contactin-associated protein-like 2 (CASPR2) protein are shown separately. Diagram not perfectly to scale. Works cited are listed in Supplementary Material 1. ASD, autism spectrum disorder.

**FIGURE 3:**
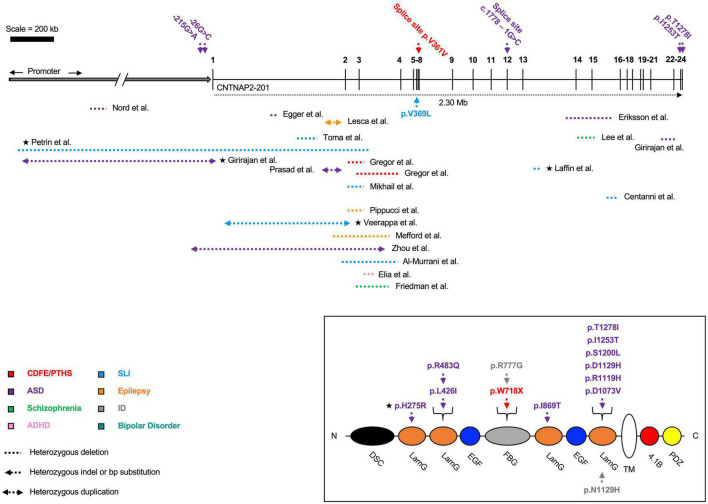
Heterozygous contactin-associated protein-like 2 (*CNTNAP2*) mutations. Mutations affecting the *CNTNAP2* locus are shown with their associated disorders. Mutations in patients with multiple affected genes are marked by a star (★). Autism spectrum disorder (ASD) is the most common diagnosis, though patients typically fall into multiple categories. The majority of deletions are found before exon 8. Mutations that map to a subdomain of the contactin-associated protein-like 2 (CASPR2) protein are shown separately. Diagram not perfectly to scale. Works cited are listed in Supplementary Material 2. CDFE/PTHS (cortical dysplasia focal epilepsy); ADHD (attention deficit hyperactivity disorder); SLI (specific language impairment); ID (intellectual disability).

Homozygous loss-of-function mutations usually lead to a diagnosis of either cortical dysplasia focal epilepsy (CDFE) or Pitt-Hopkins syndrome (PTHS) (Zweier et al., 2009; Freri et al., 2021). Both disorders are characterized by uncontrollable seizures, language regression, social/behavioral disturbances, and intellectual disability (Figure 2 and Table 1). Most *CNTNAP2* mutations are heterozygous, suggesting two functional copies of the gene are required for normal cognitive function (Figure 3). Homozygous mutations cause the most severe phenotypes and are often found in children of unaffected carrier parents (Strauss et al., 2006). This implies certain *CNTNAP2* mutations are fully penetrant while others are not.

**TABLE 1:** Homozygous contactin-associated protein-like 2 (*CNTNAP2*) mutations.

Mutation	L39X	del33-500	A156X	S329X	E494X	del1498-1671	c.1777 + 2T > C	C682X	I1253X
*Mutation location and size*	Exon 2–3; 203 kb	Exons 2–9; 1.15 Mb	Exon 3–intron 3; 76.8 kb	Exon 7; 1 bp (c.985delA)	Exon 9; 1 bp (c.1480G > T)	Exon 10; 173 bp	Exon 11–intron 11; 1 bp	Exon 13; 1 bp (c.2046C > A)	Exon 22; 1 bp (c.3709delG)
*Mutation effect*	Frameshift, premature stop	Loss of functional domains	Frameshift, premature stop	Frameshift, premature stop	Premature stop	Loss of exon 10	Splice site disruption	Premature stop	Frameshift, premature stop
*No. of patients*	2	2	2	1	1	2	1	2	9
*Diagnosis*	–	PTHS	–	–	–	–	–	–	CDFE
*Patient sex*	F, F	M, F	M, F	M	F	M, M	M	M, M	–
*Parents*	Healthy carriers	Healthy carriers	Healthy carriers	Healthy carriers	Healthy carriers	–	–	Healthy carriers	–
*ID*	Severe	Severe	Severe	Moderate	Severe	Severe	Severe	Severe	Severe
*Speech*	No	No	Simple (F), no (M)	No	No	–	–	No	Yes, with regression
*Walking*	–	Normal	Delayed (4 yrs.)	Delayed (30 mo.)	Delayed (2 yrs.)	–	–	No	Delayed (16–30 mo.)
*Age of seizure onset*	20–36 mo.	22–30 mo.	2 yrs.	14 mo.	2 yrs.	–	16 mo.	2 yrs.	14–20 mo.
*Autistic features*	Yes	No	–	Yes	Yes	–	–	Yes	Yes (67%)

Reported homozygous mutations affecting the CNTNAP2 locus. All patients develop severe seizures in infancy, language impairment, and intellectual disability (ID). Double dashes indicate data was not reported. Source publications as follows: L39X = Rodenas-Cuadrado et al. (2016); del33-500 = Zweier et al. (2009); A156X = Watson et al. (2014); S329X = Riccardi et al. (2019); E494X = Smogavec et al. (2016); del1498-1671 = Karaca et al. (2015); c.1777 + 2T > C = Parrini et al. (2017); C682X = Smogavec et al. (2016); I1253X = Strauss et al. (2006), Jackman et al. (2009).

In addition to the association between mutation homozygosity and disease severity, there are other lines of evidence suggesting *CNTNAP2* dosage is important for determining phenotype. Specifically, Nord et al. (2011) identified an autism patient with a 62 kb deletion in the *CNTNAP2* promoter region. This deletion was confirmed to reduce *CNTNAP2* gene expression in patient-derived lymphoblasts (versus those from healthy controls). A separate study from Chiocchetti et al. (2015) found a novel variant, g.-215G > A, associated with ASD and delayed age of first word. The variant also lies within the *CNTNAP2* promoter and was predicted to disrupt transcription factor binding sites. In a luciferase assay, the g.-215G > A variant was shown to significantly decrease enhancer potential in SH-SY5Y cells.

Intronic disease-causing mutations in *CNTNAP2* have also been discovered. Three unrelated patients with deletions in intron 1, which contains a regulatory element that binds with the FOXP2 transcription factor, displayed dysarthric language, autism, intellectual disability, bipolar disorder and/or ADHD (Rodenas-Cuadrado et al., 2014; Scala et al., 2021). Thus, changes in the expression level of the gene itself are sufficient to cause disorder and may explain the emergence of specific brain phenotypes.

### Contactin-associated protein-like 2 may promote neurite development

Dendritic abnormalities have been noted in humans harboring *CNTNAP2* mutations. Post-mortem brain analyses of *CNTNAP2* patients with CDFE have identified neurons in the temporal cortex with irregularly oriented dendritic processes (Strauss et al., 2006). While no additional studies in human patients are available, there is strong evidence in mice to suggest *Cntnap2* is involved in neurite development and/or synaptic transmission.

Anderson et al. (2012) showed shRNA-mediated knockdown of CASPR2 in mouse cortical cultures decreased the length and branching of neurites in excitatory neurons. These effects caused a reduction in the amplitude and frequency of excitatory and inhibitory mini post-synaptic currents. Delivery of the CASPR2 shRNA with either lentivirus or calcium phosphate transfection – targeting all neurons or individual neurons, respectively – both produced the same results, suggesting the observed effects were cell-autonomous. No changes to dendritic spine density or synapse density were observed, however, the width of spine heads was significantly reduced.

A subsequent study by Canali et al. (2018) examined neuronal cultures from both homozygous and heterozygous knockout mice. Homozygous knockout neurons had significantly reduced axon lengths, while heterozygous neurons were intermediate to wild type and homozygous. Recent work by Elia et al. (2010) came to similar findings. They over-expressed *CNTNAP2* p.R777G, a mutation associated with intellectual disability and epilepsy, in mouse cortical neurons. Both neurite branching and neurite outgrowth were decreased in the mutant cultures. These neurons had reduced amplitude of spontaneous excitatory post-synaptic currents (EPSCs), in addition to decreased action potential firing. Such reductions in EPSC amplitude have also been reported by other groups (Fernandes et al., 2019; Kim et al., 2019; Lazaro et al., 2019; Sacai et al., 2020).

Finally, Gao et al. (2018) reported that *Cntnap2* is involved in neurite outgrowth, but only in cortical interneurons. The authors showed mature interneurons from *Cntnap2* knockout mice (*in vitro* and *in vivo*) had reduced dendritic branching and dendritic length. No phenotype was observed in excitatory neurons from the same knockout mice or cell cultures. Moreover, as no difference in branching or length was observed in immature neurons (in either inhibitory or excitatory cells), the authors concluded the reduction in branching was due to decreased neurite stabilization and not impaired outgrowth.

### Contactin-associated protein-like 2 may modulate dendritic spine density

As well as the proposed role for *Cntnap2* in neurite branching and stabilization, several studies have suggested the gene may regulate dendritic spine dynamics. Gdalyahu et al. (2015) were the first to report a reduction in dendritic spine density in an *in vivo* study of Thy1-GFP/*Cntnap2* null mice. Using 2-photon microscopy, the authors showed knockout mice had significantly decreased spine density in cortical layer Vb. The reduction was caused by decreased stability of newly formed spines (i.e., loss of spines shortly after they form). No reduction in the formation of new spines was observed, nor was any effect on the maintenance or pruning of already-formed/stable spines. This suggests *CNTNAP2* may be required for the stabilization of new synaptic contacts, a process thought to be critical for brain plasticity (Gdalyahu et al., 2015).

Lazaro et al. (2019) added further evidence that loss of *Cntnap2* reduces dendritic spine density *in vivo*. The authors reported knockout mice had significantly decreased spine densities and synaptic inputs in layer II–III excitatory neurons. This resulted in a 2-fold decrease in the frequency and amplitude of mEPSCs. No differences in intrinsic neuronal excitability, neurotransmitter release probability, or synapse maturity were observed between genotypes. The knockout neurons did, however, have reduced network synchrony and less precise firing patterns.

Lastly, Varea et al. (2015) have also reported reductions in dendritic spine density in *in vitro* knockout cultures. The authors additionally observed reduced GluA1 AMPA receptor subunit expression in spines, and GluA1 cytoplasmic aggregates in cell bodies. These aggregates were found to contain trafficking proteins (e.g., clathrin and rab5), suggesting loss of *Cntnap2* could affect intracellular GluA1 transport. Two other papers reported similar reductions in glutamate receptor expression (Fernandes et al., 2019; Kim et al., 2019). More recent work identified that CASPR2 binds GluA1 through complexing with the protein CASK (Gao et al., 2019). Mutations in the gene encoding CASK have been linked to intellectual disability and ASD, which implies the pathway downstream of the CASPR2-CASK complex is important for disease pathophysiology (Becker et al., 2020).

### Contactin-associated protein-like 2 and cortical interneurons

Contactin-associated protein-like 2 is robustly expressed in interneurons, and in the ganglionic eminence where interneurons derive from Peñagarikano et al. (2011); Gordon et al. (2016). Peñagarikano et al. (2011) described a loss of GABAergic interneurons in *Cntnap2* null mice. The authors noted a significant reduction in all cortical layers. Parvalbumin positive (PV^+^) interneurons were the most affected (20% loss), while calretinin- (CALB2) and neuropeptide Y- (NPY) positive neurons were also significantly reduced. The loss of interneurons was hypothesized to underly the frequent seizures observed in *Cntnap2* null mice (as reported by others, see Hoffman et al., 2016; Thomas et al., 2017). *In vivo* 2-photon calcium imaging of layer II–III neurons revealed firing was highly asynchronous relative to wild type. The authors did not detect any changes to firing amplitude or frequency, suggesting the asynchronicity was not due to abnormal neuronal activity/conduction, but to defects in synaptic networks.

These findings were followed up by a study from Selimbeyoglu et al. (2017), who found that the PV^+^ interneurons of *Cntnap2* knockout mice had significantly decreased activity *in vivo*. Activating PV^+^ interneurons or inhibiting excitatory neurons rescued the observed excitation: inhibition imbalance. Finally, a recent study by Hali et al. (2020) reported significant reductions in the number of interneurons in cortical organoids derived from *Cntnap2* knockout mice. No differences in glutamatergic neurons were observed. The authors also noted knockout organoids had dramatically reduced expression of transcription factors expressed in ventral telencephalic (interneuron) progenitor cells (e.g., *Dlx2*, *Nkx2.1*, and *Ascl1*). Similar results have also been observed in a zebrafish *Cntnap2* knockout model (Hoffman et al., 2016) and in *Cntnap2* knockout mouse hippocampal neurons (Paterno et al., 2021).

### Contactin-associated protein-like 2 and human evolution

#### What are human accelerated regions?

**FIGURE 4:**
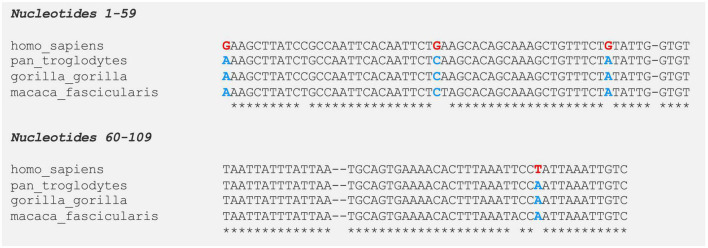
Multiple species alignment of human accelerated conserved non-coding sequence 97 (HACNS_97) [109 bp HAR located within intron 1 of contactin-associated protein-like 2 (*CNTNAP2*)]. Human accelerated regions (HARs) are sequences of DNA that are highly conserved in non-human primates but have an unexpected number of unique nucleotide substitutions in humans. On average, a 100 bp-long HAR will contain ∼1.7 human-specific substitutions (HARs have a mean length of 266 bp). In contrast, chimpanzees (who carry the highly conserved ortholog), will have ∼0.2 unique substitutions. Even if a HAR gains only a small number of human-specific changes, this rate is significantly higher than observed in other conserved elements. Moreover, the regions surrounding HARs are usually still conserved, suggesting HARs may be part of a larger functional structure. Human Accelerated Conserved Non-coding Sequence 97 (HACNS_97) is shown as an example. HACNS_97 contains four nucleotides that are fixed in all humans but are absent in other primates (nucleotides highlighted in red and blue, respectively). *Pan troglodytes* = chimpanzee; *Gorilla gorilla* = western gorilla; *Macaca fascicularis* = crab-eating macaque.

The sheer size of the human genome (approximately 3 billion nucleotides) poses a major challenge for identifying the genomic regions important for human evolution. In order to prioritize sequences for further study, Pollard et al. (2006) published a statistical test to find “HARs.” As mentioned in the introduction of this paper, HARs are DNA sequences that fulfill two key criteria (see Figure 4; Pollard et al., 2006; Prabhakar et al., 2006; Bird et al., 2007; Bush and Lahn, 2008; Lindblad-Toh et al., 2011):

(i) They are highly conserved across a wider clade (e.g., primates, mammals, or vertebrates) – this suggests the region may be functional;
(ii) They have an unexpectedly large number of human-specific nucleotide changes – this suggests the sequence may be important for human evolution.

Approximately 4,000 HARs have been identified to date – ∼97% of them in non-coding portions of the genome (Capra et al., 2013; Uebbing et al., 2021). This distribution has made it difficult to assign HARs a function, because most of the non-coding genome is uncharacterized. Even so, there is growing evidence that many HARs are gene enhancers. For example, Capra et al. (2013) used existing functional genomics data, in combination with machine learning algorithms, to show that 60% of non-coding HARs overlap epigenetic enhancer marks like H3K4me1, H3K27ac, or p300. Half of these HARs were predicted to target genes active during development, and one third to act in the brain.

These predictions were supported by the finding that HARs are highly enriched in transcription factor binding motifs (Capra et al., 2013; Doan et al., 2016). ∼60% of HARs are also located within 1 Mb of a gene that is differentially expressed between humans and chimpanzees (Levchenko et al., 2018). While only a few HARs have been experimentally characterized (most using mouse enhancer assays), these studies have generally validated the predictions made by Capra et al. (2013) (Ryu et al., 2018; Girskis et al., 2021; Uebbing et al., 2021).

#### Contactin-associated protein-like 2 contains eight human accelerated regions

Contactin-associated protein-like 2 is unusual for containing eight HARs in its introns (Table 2):

**TABLE 2:** Contactin-associated protein-like 2 (*CNTNAP2*) human accelerated regions (HARs).

HAR	Intron	Coordinates	Length (bp)	No. of human substitutions	No. polymorphic	No. shared with archaic humans	No. A/T to G/C
*HACNS_116 (Prabhakar)*	1	chr7: 146,214,973–146,215,168	196	5	1	5	2
*HACNS_97 (Prabhakar)*	1	chr7: 146,290,445–146,290,553	109	4	0	4	2
*2xHAR.395 (Linblad-Toh)*	1	chr7: 146,420,329–146,420,351	24	3	0	3	2
*HACNS_884 (Prabhakar)*	1	chr7: 146,654,063–146,654,409	347	5	0	5	3
*ANC1208* *(Bird)*	11	chr7: 147,516,200–147,516,488	289	4	0	3	1
*HACNS_590 (Prabhakar)*	13	chr7: 147,859,118–147,859,418	301	4	0	4	1
*ANC1209* *(Bird)*	13	chr7: 147,878,720–147,878,918	199	4	0	2	1
*HACNS_954 (Prabhakar)*	18	chr7: 148,173,396–148,173,905	510	6	0	6	3

Eight HARs lie within the CNTNAP2 locus. Of these, four are located within intron 1 and two are located in intron 13. The number of human-specific nucleotide substitutions is shown in column 5, followed by the number of these substitutions that are polymorphic in humans (column 6), the number shared in Neanderthals/Denisovans (column 7), and the number of substitutions that are G/C in humans from A/T in other primates (i.e., weak to strong). The majority of CNTNAP2 HARs appear to be composed of fixed substitutions that are shared with archaic humans. Between 1/4 and 1/2 are weak to strong transitions. Coordinates map to human genome GRCh38; original sources describing each HAR are shown in brackets. Data adapted from Hubisz and Pollard (2014).

1. HACNS_116^1^ (Prabhakar et al., 2006);
2. HACNS_97 (Prabhakar et al., 2006);
3. 2xHAR.395 (Lindblad-Toh et al., 2011);
4. HACNS_884 (Prabhakar et al., 2006);
5. ANC1208^2^ (Bird et al., 2007);
6. HACNS_590 (Prabhakar et al., 2006);
7. ANC1209 (Bird et al., 2007);
8. HACNS_954 (Prabhakar et al., 2006).

While this is likely related to the sheer size of the gene, the density of HARs is still higher than expected. Since *CNTNAP2* is 2.3 Mb long, one would expect only two to three HARs to fall within the gene (using the 4,000 HARs identified, and assuming that HARs are evenly distributed across the genome). From this perspective, eight HARs would be unexpectedly high.

Half of the *CNTNAP2* HARs are located in intron 1 – a common location for gene regulatory elements (Chorev and Carmel, 2012). The HARs range in length from 24 bp (2xHAR.395) to 510 bp (HACNS_954), and from 3 to 6 human-specific substitutions. Most of these human-specific changes are shared with Neanderthals and Denisovans, indicating they arose before the emergence of *Homo sapiens*. However, HACNS_97, ANC1209, ANC1209, and HACNS_954 each contain one substitution that is unique to modern humans alone (Burbano et al., 2012). Multiple species alignments for each *CNTNAP2* HAR can be found in Figure 4 and Supplementary Figures 1–7.

Although HARs are a highly powerful tool, a number of caveats apply to their interpretation. These include the low reproducibility between studies that have used different methods to detect HARs, and the dependence on the size of human reference panels in defining which mutations are likely to be fixed in humans species-wide. Each of the *CNTNAP2* HARs was identified by only one of the six major HAR publications [excluding HACNS_884, which was identified by both Gittelman et al. (2015) and Prabhakar et al. (2006)]. Secondly, one of the HARs, HACNS_116, has a human-specific substitution that was subsequently found to be polymorphic (Hubisz and Pollard, 2014). All remaining HAR substitutions appear – according to currently available evidence – to be fixed in humans.

That said, there is some indication that one or more of the *CNTNAP2* HARs could be enhancers. HACNS_884 was shown by Gittelman et al. (2015) to overlap a human-specific DNase I hypersensitive site (DHS). DNase I selectively cleaves regions of open/active DNA, which is the expected chromatin state of regulatory elements (Dorschner et al., 2004). Won et al. (2019) further identified six of the eight HARs as overlapping DHSs in fetal brain (all except 2xHAR.395 and ANC1208). Similarly, Capra et al. (2013) detected HACNS_884 and HACNS_954 as putative enhancers using their enhancer finding pipeline. They were also able to bioinformatically predict that HACNS_884 is active in fetal brain but could not provide a prediction for HACNS_954. More recently, Girskis et al. (2021) identified HACNS_116, HACNS_590, and ANC1209 as potential enhancers using a massively parallel reporter assay. Interestingly, HACNS_116 was found to have over 2x the enhancer activity as its chimpanzee ortholog.

**FIGURE 5:**
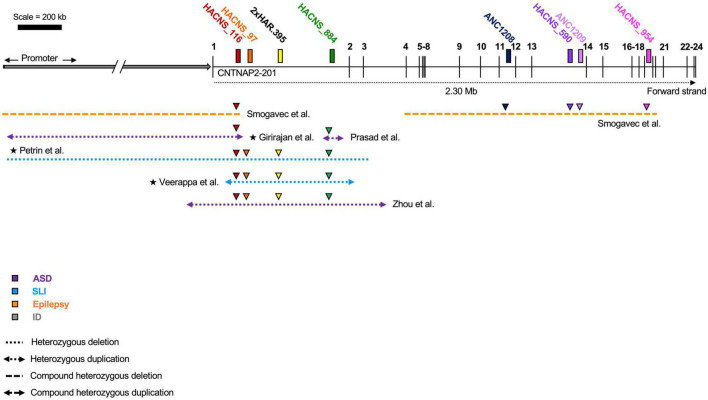
Overlap of human accelerated regions (HARs) and contactin-associated protein-like 2 (*CNTNAP2*) mutations. Disease-associated mutations that overlap one or more human accelerated region. Colored triangles indicate overlap: the color represents the specific HAR involved. Mutations in patients with multiple affected genes are marked by a star (★). HACNS_116 in intron 1 intersects the most mutations (five), while ANC1208, HACNS_590, ANC1209, and HACNS_954 all intersect the fewest (one mutation). ASD (autism spectrum disorder); SLI (specific language impairment); ID (intellectual disability). Diagram not perfectly to scale.

Also suggestive of their functional role is the fact that the *CNTNAP2* HARs overlap deletions associated with neurodevelopmental disorders, including ASD, SLI, ID, and epilepsy (Figure 5). Curiously, all of these disease-linked mutations are heterozygous (or compound heterozygous), which suggests that the loss of even a single copy of a HAR may be enough to affect brain function. However, there is no evidence to suggest directly that these mutations are pathogenic due to the loss of the HAR. This cannot be assumed, particularly because many of the mutations are large, and therefore not specific. Moreover, most of the mutations encompass multiple HARs, suggesting it could be a combinatorial loss of HAR function(s) that leads to disease. HACNS_116 overlaps the most pathogenic mutations (five in number), followed by HACNS_884 (four in number), and then HACNS_97 and 2xHAR.395 each with three.

### Contactin-associated protein-like 2 contains signatures of positive selection in human populations

A “selective sweep” occurs when a positively selected variant rapidly increases in frequency, and nearby linked variants rise in frequency along with it (Jobling, 2014). This occurs as there is no time for recombination to break down the linkage between the selected and non-selected loci. In other words, sweeps cause genetic diversity in the region around the selected variant to decrease (Sabeti et al., 2006).

Evidence for selective sweeps have been identified at the *CNTNAP2* locus in humans (Ayub et al., 2013; Mozzi et al., 2016). Specifically, Ayub et al. (2013) detected sweep signatures in *CNTNAP2* introns 1 and 13. Since Neanderthals and Denisovans carry the ancestral sequences at these loci, this suggests selection began after the split of modern humans from archaic hominins. The sweep signatures were identified in some but not all human populations, implying that selection occurred after the Out-of-Africa (OOA) dispersals. Introns 1 and 13 each contain several HARs: HACNS_116, HACNS_97, 2xHAR.395, and HACNS_884 in intron 1, and HACNS_590 and ANC1208 in intron 13. Notably, HACNS_97 coincides directly with one of the sweeps and may therefore be a target for further study.

## Conclusion

Taken altogether, there are initial grounds to suggest *CNTNAP2* has played a role in the evolution of the human brain. There is also a strong body of literature linking mutations in *CNTNAP2* to neurodevelopmental disorders. Despite these advances, a number of unanswered questions remain about *CNTNAP2* function, and in particular, the mechanism(s) through which *CNTNAP2* acts.

**FIGURE 6:**
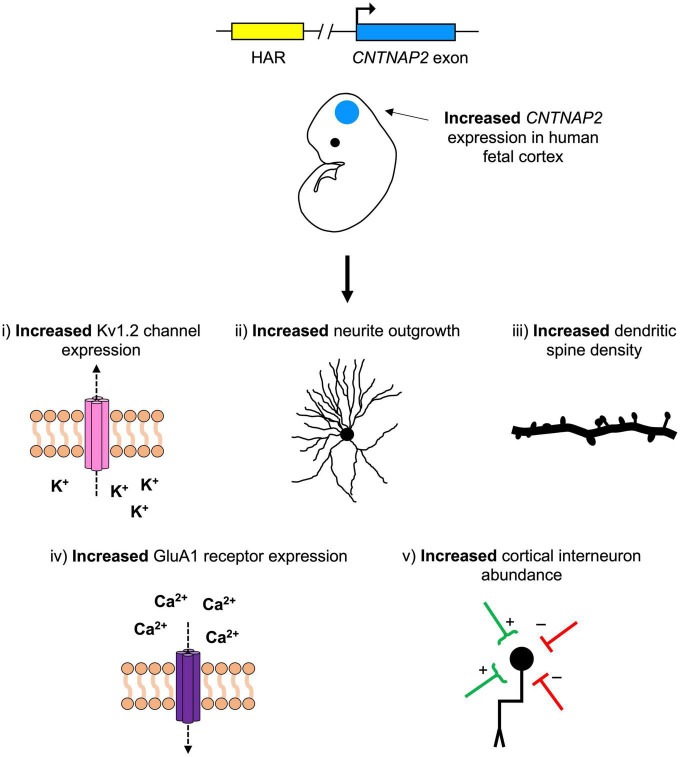
A hypothetical model for the role of contactin-associated protein-like 2 (*CNTNAP2*) in human neurodevelopmental disease and cerebral cortex evolution. Mutations that decrease *CNTNAP2* expression [e.g., nonsense mutations or those disrupting a human accelerated region (HAR)] cause reductions in (1) potassium channel (Kv1.2) expression, (2) neurite branching, (3) dendritic spine density, (4) glutamate receptor (GluA1) expression, and (5) cortical interneuron abundance. Since *CNTNAP2* has higher expression in the cortex of humans than in non-human primates, the opposite (i.e., increases in these features) may have contributed to differences in human versus non-human primate brain function. One (or more) of the *CNTNAP2* HARs may be driving this increase in expression by functioning as an enhancer with human-specific properties.

One attractive hypothesis is that one or more of the *CNTNAP2* HARs are gene enhancers. These may be driving human-specific patterns of *CNTNAP2* expression in the developing cortex. In turn, this human-specific increase in *CNTNAP2* expression may have contributed to human brain function and increased human susceptibility to cognitive disorders. The studies summarized in this review suggest five mechanisms by which this may have occurred: increases in (1) potassium channel expression, (2) neurite development, (3) dendritic spine formation, (4) glutamate receptor expression, and/or (5) cortical interneuron abundance (Figure 6). Further experimentation is needed to clarify which of these mechanisms (if any) are at play, and which genes/pathways they involve.

Additionally, little experimental work has been conducted in human models, with most studies limited to either rodents or zebrafish. Human systems should be adopted for investigations into *CNTNAP2* function, including the use of stem cell-derived neurons and primary patient samples, where possible. Such studies will be invaluable for understanding the role of *CNTNAP2* in human-specific neurobiology, and in deciphering the downstream molecular events caused by human *CNTNAP2* mutations.

## Author contributions

FS researched and wrote the manuscript under the supervision of FL and TK. All authors contributed to the article and approved the submitted version.

## Footnotes

1 HACNS: human accelerated conserved non-coding sequence.

2 ANC: accelerated conserved non-coding sequence.
