# Supplementary material for: The role of contactin-associated protein-like 2 in neurodevelopmental disease and human cerebral cortex evolution
Source: Front Mol Neurosci. 2022 Oct 20;15:1017144. doi: 10.3389/fnmol.2022.1017144 (PMC9630569; doi:10.3389/fnmol.2022.1017144)
Supplement: Supplementary file 1 [file Data_Sheet_1.PDF]

```
Nucleotides 1-60
homo_sapiens          GGAACATTTGGCTTAAAGTTTCAGAAAATCTGCTCCGTTTGATAATGTCAACTCATCACA
pan_troglodytes       GGAACATTTGGCTTAAAGTTTCAGAAAATCTGCTCAGTTTGATAATGTCAACTCATCACA
macaca_fascicularis   GGAACATTTGGCTTAAAGTTTCAGAAAATCTGCTCAGTTTGATAATGTCAACTCATCACA
                      *********************************** ************************
Nucleotides 61-120
homo_sapiens          TTTCAGTTAAGCAGACATCAAATCAAATGACTCATGTCACAGATACATAAATATTACGTA
pan_troglodytes       TTTCAGTTAAGCAGACATCAAATCAAATGATGCATGTCACAGATACATAAATATTACGTA
macaca_fascicularis   TTTCAGTTAAGCAGGCATCAAATCAAATGATGCATGTCACAGATACATAAATATTATGTA
                      ************** ***************  ************************ ***
Nucleotides 121-180
homo_sapiens          CATGGAAAGGAAATTTTTAGCTATAAATGCATAAAATCTTCTGGTTTGTAAACCTGAACT
pan_troglodytes       CATGGAAAGCAAATTTTTAGCTATAAATGCATAGAATCTTCTGGTTTGTAAACCTGAACT
macaca_fascicularis   CATGGAAAGCAAATTTTTAGCTATAAATGCATAGAATCTTCTGGTTTGTAAACCTGAACT
                      ********* *********************** **************************
Nucleotides 181-196
homo_sapiens          ACATTTTAAAATTTTG
pan_troglodytes       ACATTTTAAAATTTTG
macaca_fascicularis   ACATTTTAAAATTTTG
                      ****************
```

**Supplementary Figure 1. Multiple species alignment of HACNS_116 (196 bp HAR located within intron 1 of *CNTNAP2*)**

Human Accelerated Conserved Non-coding Sequence 116 (HACNS_116) contains five nucleotides that are fixed in all humans but are absent in other primates (nucleotides highlighted in red and blue, respectively). *H. sapiens* = human; *P. troglodytes* = chimpanzee, *M. fascicularis* = crab-eating macaque.

```
Nucleotides 1-24

homo_sapiens           AAGGGTTTCTGTTAATGAACAAAG
pan_troglodytes        AAGTGCTTCTTTTAATGAACAAAG
gorilla_gorilla        AAGTGCTTCTTTTAATGAACAAAG
macaca_fascicularis    AAGTGCTTCTTTTGATGAACAAAG
                       *** * **** ** **********
```

**Supplementary Figure 2. Multiple species alignment of 2xHAR.395 (24 bp HAR located within intron 1 of *CNTNAP2*)**

Human Accelerated Region 395 (2xHAR.395) contains three nucleotides that are fixed in all humans but are absent in other primates (nucleotides highlighted in red and blue, respectively). *H. sapiens* = human; *P. troglodytes* = chimpanzee, *G. gorilla* = western gorilla; *M. fascicularis* = crab-eating macaque.

***Nucleotides 1-75***

```
homo_sapiens           TGTAAAGCTGAGGCCCAGGAGAAGCTCTTTTTC-----TTACCCATTTGGTCTTTAATGTTGCTTGCGAATGCATGGGGG
pan_troglodytes        TGTAAAGCTGAGGCCCAGGAGAAACTCTTTTTC-----TTACCCATTTGGTCTTTAATGTTGCTTGCGAATGCATGGGGG
macaca_fascicularis    TATAAAGCTGAGGCCCAGGGGGAGGTCTTTTTC-----CCACCCATTTGGTTTTTAATGTTGCTTGTGAATGCATGGGGG
                       * ***************** * *  ********       *********** ************** *************
```

***Nucleotides 76-154***

```
homo_sapiens           TAGAACTCTAATAACAATGATGGACACGATGTCCTTGCACATAAACATTTGGCTCCTCACAGCATGAGATGTCTGGTCA
pan_troglodytes        TAGAACTCTAATAACAATAATGGACACAATGTCCTTGCACATAAACATTTGGCTCCTCACAGCATGAGATGTCTTGTCA
macaca_fascicularis    TAGAACTCTAATAACAATAATGGACACGATGTCCTTGCACATAAACATTTGGCTCCTCACAGCGTGAGATGTCTTGTCA
                       ****************** ******** *********************************** ********** ****
```

***Nucleotides 155-232***

```
homo_sapiens           GCCACAGAGGATAAAATAATGGCGTAAAGATTTACTGTCAGGGAGCTGGGCCAGTCTCAGCAGGACAGCGGCTCCAAG
pan_troglodytes        GCCACAGAGGATAAAATAATGGCGTAAAGATTTACTGTCAGGGAGCTGGGCCAGTCTCAGCAGGACGGCGGCTCCAAG
macaca_fascicularis    GCCACAGAGGATAAAATAATGGCGTAAAGATTTACTGTCAGGGAGCTGGGCCAGTCTCAGCAGGATGGCAGCTCCAAG
                       *****************************************************************  ** ********
```

***Nucleotides 233-308***

```
homo_sapiens           CAGATAGATGGC-CTGGTATGCGTAGGTCACCTGTGTTTACACTGATGAATGTGTGTGGTTTCCCTAGTCTGCATCA
pan_troglodytes        CAGATAGATGGC-CTGGTATACGTAGGTCACCTGTGTTTACACTGACGAATGTGTGTGGTTTCCCTAGTCTGCATCA
macaca_fascicularis    CAGATAGATGGC-CTGGCATGCGTAGGTCACCTGTGTTTACACTGAGGAATGTGTGTGGTTTCCCTAGTCTGCATCA
                       ************ **** ** ************************* ******************************
```

***Nucleotides 309-347***

```
homo_sapiens           TTATATTGAGTTGTGTGGTGTTGGTAGCACTTGTTACTC
pan_troglodytes        TTATATTGAGTTGTGTGGTGTTTGTAGCACTTGTAACTC
macaca_fascicularis    TTATATTGAGTTGTGTGGTGTTTGTAGCACTTGTAACTC
                       ********************** *********** ****
```

**Supplementary Figure 3. Multiple species alignment of HACNS_884 (347 bp HAR located within intron 1 of *CNTNAP2*)**

Human Accelerated Conserved Non-coding Sequence 884 (HACNS_884) contains five nucleotides that are fixed in all humans but are absent in other primates (nucleotides highlighted in red and blue, respectively). *H. sapiens* = human; *P. troglodytes* = chimpanzee, *M. fascicularis* = crab-eating macaque.

```
Nucleotides 1-68
homo_sapiens            TTA---AAAGTTGTCTAT--TGCTAATTATATATTT-GTACTT----TT-TTTTA-CAGCTACTCATGTGTAGAACAAAA
pan_troglodytes         TTA---AAAATTGTCTAT--TGCTAATTATATATTT-GTACTT----TT-TTTTA-CAGCTACTCATGTGTAGAACAAAA
gorilla_gorilla         TTA---AAAATTGTCTAT--TGCTAATTATATATTT-GTACTT----TT-TTTTA-TAGCTACTCATGTGTAGAACAAAA
macaca_fascicularis     ---ATAAAAATTA----T--TGCTAATTATATATTT-GTACTTGCTTTT-TTTTA-TAGCTACTCATGTGTAGAACAAAA
                              *** **     *  **************** ******    ** *****  ***********************
Nucleotides 69-138
homo_sapiens            AAATA--GACAAAATTACTCA--GGCTCAATGATTCACTATGTACAGTTTACAAATTAATT----AGCTT-CTCTTAAT
pan_troglodytes         AAATA--GACAAAATTACTCA--GGCTCAATGATTCACTATGTACAGTTTACAAATTAATT----AGCTT-CTCTTAAT
gorilla_gorilla         AAATA--GACAAAATTACTCA--GGCTCAATGATTCACTATGTACAGTTTACAAATTAATT----AGCTT-CTCTTAAT
macaca_fascicularis     AAATA--GACAAAATTAATCATAGGCTCAATGATTCACTATGTACAGTTTACAAATT----AATTAGCTT-CTCTTAAT
                        *****  ********** ***  **********************************        ***** ********
Nucleotides 139-216
homo_sapiens            TTTTTCAATTAAAAAAGTGTTAAAGCATTAGTTTATTGCTTATTTCCGTGTTAAAATGCTGGTTAATTGCAAAACATT
pan_troglodytes         CTTTTCAATTAAAAAAGTGTTAAAGCATTAGTTTATTGCTTACTTCCGTGTTAAAATGCTGGTTAATTGCAAAACATT
gorilla_gorilla         CTTTTCAATTAAAAAAGTGTTAAAGCATTAGTTTATTGCTTACTTCCGTGTTAAAATGCTGGTTAATTGCAAAACATT
macaca_fascicularis     CTTTTCAATTAAAAAAGTGTTAAAGCATTAGTTTATTGCTTACTTCCGTGTTAAAATGCTGGTTAATTGCAAAACATT
                         ***************************************** ***********************************
Nucleotides 217-289
homo_sapiens            ATTAGGGGCCAATATTAAAATTCCACTTACAAGTGTCAGAGAAGCAAAACACATTTAAAGTTCATTTGCAAAT
pan_troglodytes         ATTAGGGGCCAATATTAAAATTCCACTTACAAGTGTCAGAGAAGCAAAACACATTTAAAGTTGATTTGCAAAT
gorilla_gorilla         ATTAGGGGCCAATATTAAAATTCCACTTACAAGTGTCAGAGAAGCAAAACACATTTAAAGTTGATTTGCACAT
macaca_fascicularis     ATTAGGGGCCAATATTAAAATTCCACTTACAAGTGTCAGAGAAGCAAAACACATTTAAAGTTGATTTGCAAAT
                        ************************************************************** ******* **
```

**Supplementary Figure 4. Multiple species alignment of ANC_1208 (289 bp HAR located within intron 11 of *CNTNAP2*)**

Accelerated Conserved Non-Coding Sequence 1208 (ANC_1208) contains four nucleotides that are fixed in all humans but are absent in other primates (nucleotides highlighted in red and blue, respectively). *H. sapiens* = human; *P. troglodytes* = chimpanzee, *G. gorilla* = western gorilla; *M. fascicularis* = crab-eating macaque.

***Nucleotides 1-75***

```
homo_sapiens          AAGATGATTTTTT-CAAGCATATGTTACCAAGAAGTAGG----GAAGTTTCAGCATTAACA--ATACATAGCTTCGTAACAA
pan_troglodytes       AAGATGATTTTTT-CAACCATATGTTACCAAGAAGTAGG----GAAGTTTCAGCATTCACA--ATACATAGCTTCGTAACAA
gorilla_gorilla       AAGATGATTTTTT-CAACCACATGTTACCAAGAAGTAGG----GAAGTTTCAGCATTAACA--ATACATAGCTTCGTAACAA
macaca_fascicularis   AAGATGATTTTTT-CAACCATATGTTACCAAGAAGTAGG----GAAGTTTCAGCATTAACA--ATACAGAGCTTCGTAACAA
                      ************* *** ** ******************    ************** ***  ***** *************
```

***Nucleotides 76-157***

```
homo_sapiens          TTAGCCATCTGTTTATAATGCTGTTAGGGATCGACAGCATCTCAATGGAAGCAGGGAAAACAACAGAAATATCATATCTGCC
pan_troglodytes       TTAGCCATCTGTTTATAATGCTGTTAGGGATCGACAGCATCTCAATGGAAGCAGGGAAAACAACAGAAATATCATATCTGCC
gorilla_gorilla       TTAGCCATCTGTTTATAATGCTGTTAGGGATCGACAGCATCTCAATGGAAGCAGGGAAAACAACAGAAATATCATATCTGCC
macaca_fascicularis   TTAGCCATCTGTTTATAATGATGTTAGGGATTGACAGCATCTCAATGGAAGCAGGGAAAACAACAGAAATATTATATCTGCC
                      ******************** ********** **************************************** *********
```

***Nucleotides 158-239***

```
homo_sapiens          AAGTTCTAGTCATCTGTTATGTTACATAGTAATTTGTCATCTCATGAGATCACCAAGGAGAGAAAAAAGCCATGCCGTGCTA
pan_troglodytes       AAGTTCTAGTCATTTGTTATCTTACATAGTAATTTGTTGTCTCATGAGATCACCAAGGAGAGGAAAAAGCCATGCCGTGCTA
gorilla_gorilla       AAGTTCTAGTCATTTGTTATCTTACATAGTAATTCGTCGTCTCATGAGATCACCAAGCAGAGAAAAAAGCCATGCCGTGCTA
macaca_fascicularis   AAGTTCTAGTCATTCGTTATCTTACATAGTAATTTGCTGTCTCATGAGATCACTAAGGAGAGAAAAACACCATGCCGTGCTA
                      *************  ***** ************* *   ************** *** **** ****  *************
```

***Nucleotides 240-301***

```
homo_sapiens          CAAAATAAACTCTGTAAAGCAGGTAGTTTAATAGCAAATTAATGATGTGCTGACAAGATCAA
pan_troglodytes       CAAAATAAACTCTGTAAAGCAGGTAGTTTAATAGCAAATTAATGATGTGCTGACAAGATCAA
gorilla_gorilla       CAAAATAAACTCTGTAAAGCAGGTAGTTTAATAGCAAATTAATGATGTGCTGACAAGATCAA
macaca_fascicularis   CAAAATAAACTTTGTAAAGCAGGTAGTTTAATAGCAAATTAATGATGTGCTGACAAGATCAA
                      *********** **************************************************
```

**Supplementary Figure 5. Multiple species alignment of HACNS_590 (301 bp HAR located within intron 13 of *CNTNAP2*)**

Human Accelerated Conserved Non-coding Sequence 590 (HACNS_590) contains four nucleotides that are fixed in all humans but are absent in other primates (nucleotides highlighted in red and blue, respectively). *H. sapiens* = human; *P. troglodytes* = chimpanzee, *G. gorilla* = western gorilla; *M. fascicularis* = crab-eating macaque.

```
Nucleotides 1-80
homo_sapiens          AATAAGGCAACATACATCAAATCCTTTTGAGAATATTCAACA-TGAGATAGTTTTCTTTTAAGTCTGACAGTGGCAGGCCA
pan_troglodytes       AATAAGGCAACATACATCAAATCCTTTTGAGAATATACAACA-TGAGATAGTTTTCTTTTAAGTCTGACAGTGGCAGGCCA
gorilla_gorilla       AATAAGGCAACATACATCAAATCCTTTTGAGAATATACAACA-TGAGATAGTTTTCTTTTAAGTCTGACAGTGGCAGGCCA
macaca_fascicularis   AATAAGGCAACATACATCAAATCCTTTTGAGAATATACAACA-AGAGATAGTTTTCTTTTAAGACTGATGGTGGCAGGCTG
                      ************************************ *****  ******************* ****  *********
Nucleotides 81-161
homo_sapiens          TAATACTACTGAATAAATTCTTACCAAACTGAAGCTTCTGGGCTCAGTTTCAATGTTTTATTTAGTATTGTTCTCACCAGC
pan_troglodytes       TAATACTACTGAATAAATTCTTACCAAACTGAAGCTTCTGGGCTCAGTTTCAATGTTTTATTTAGTATTATTCTCGCCAGC
gorilla_gorilla       TAATACTACTGAATAAATTCTTACCAAACTGAAGCTTCTGGGCTCAGTTTCAATGTTTTATTTAGTATTATTCTCGCCAGC
macaca_fascicularis   TAATACTACTGAATAAATTCTTACTGAACTGAAGCTTCTGGGCTCAGTTTCAATGTTTTCTTTAGTATTATTGTCGCCAGC
                      ************************  ********************************* ********* ** ** *****
Nucleotides 162-199
homo_sapiens          TTTCTTCTGACAGGGCTCAATGAAGATGTTAGTTAATA
pan_troglodytes       TTTCTTCTGACAGGGCTCAATCAAGATGTTAGTTAATA
gorilla_gorilla       TTTCTTCTGACAGGGCTCAATCAAGATGTTAGTTAATA
macaca_fascicularis   TTTCTTCTGACAGGGCTCAATCAAGATGTTAGTTAATA
                      ********************* ****************
```

**Supplementary Figure 6. Multiple species alignment of ANC_1209 (199 bp HAR located within intron 13 of *CNTNAP2*)**

Accelerated Conserved Non-Coding Sequence 1209 (ANC_1209) contains four nucleotides that are fixed in all humans but are absent in other primates (nucleotides highlighted in red and blue, respectively). *H. sapiens* = human; *P. troglodytes* = chimpanzee, *G. gorilla* = western gorilla; *M. fascicularis* = crab-eating macaque.

```
homo_sapiens         ATAAGAAGCTTTACATAAGTAGCCTGTTCAGACTCTGATGAATACATGG-AGGGTTGTTTACACTGGCATTATTCACACAAGATGCCACACACAGAA
pan_troglodytes      ATAAGAAGCTTTACATAAGTAGCCTATTCAGACTCTGATGAATACATGG-AGGATTGTTTACACTGGCATTATTCACACAAGATGCCACACACAGAA
gorilla_gorilla      ATAAGAAGCTTTACATAAGTAGCCTGTTCAGACTCTGATGAATACATGGGAGGGTTGTTTACACTGGCATTATTCACACAAGATGCCACACACAGAA
macaca_fascicularis  ATAAGAAGCTTTACATAAGTAGCCTATTCAGACTCTGATGAATACATGG-AGGGTTGTTTACACTGGCATTATTCACGCAAGATGCCACACACAGAA
                     ************************* *********************** *** *********************** *******************

homo_sapiens         GGAGGAATTGTTCCATAACATTCTCTGTACCTCCAGGCTACTGTTTGGCAGCGCTATAATATGAGCATACAAATTAGGCCAGACAGGGTATGAAAAA
pan_troglodytes      GGAGGAATTGTTCCATAACATTCTCTGTACATCCAGGCTACTGTCTGGCAGCGCTATAATATGAGCATACAAATTAGGCCAGACAGGGTATGAAAAA
gorilla_gorilla      GGAGGAATTGTTCCATAACATTCTCTGTACATCCAGGCTACTGTCTGGCAGCGCTATAATATGAGCATACAAATTAGGCCAGACAGGGTATGAAAAA
macaca_fascicularis  GGAGGAATTGTTCCATAACATTCTCTGTACATCCAGGCTACTGTCTGGCAGCGCTATAATATGAGCATACAAATTAGGCCAGACAGGGTATGAAAAA
                     ****************************** ************* ****************************************************

homo_sapiens         TGAGCATGAGACAACAACAAAGGTGGAAAGGAACACCATTGCAGAATAATACCAAAGCACATTAACAGTGCTATTACAGTGCATTTTTCTGTTCAGA
pan_troglodytes      TGAGCATGAGACAACAACAAAGGTGGAAAGGAACGCCATTGCAGAATAATACCATAGCACATTAACAGTGCTATTACAGTGCGTTTTTCTGTTCAGA
gorilla_gorilla      TGAGCATGAGACAACAACAAAGGTGGAAAGGAACACCATTGCAGAATAATACCATAGCACATTAACAGTGCTATTACAGTGCATTTTTCTGTTCAGA
macaca_fascicularis  TGAGCATGAGACAACAACAAAGGTGGAAAGGAACACTATAGCGGAATAATTCCATACCACACTAACAGCGCTATTACAGTGCATTTTTCTGTTCAGA
                     ********************************** * ** ** ******* *** * **** ****** ************* **************

homo_sapiens         ACAACATGGTTCCATGATTTTCCTGCGGCACACAATTGAATGGGCCTGCAGAACACATTAATTTTGCACTTGTAGAAATGACAGTCTTGCAATTACC
pan_troglodytes      ACAACATGGTTCCATGATTTTCCTGTGGCACACAATGGAATGGGCCTGCAGAACACATTAATTTTGCACTTGTAGAAATGACAGTCTTGCAATTACC
gorilla_gorilla      ACAACATGGTTCCATGATTTTCCTGTGGCACACAATGGAATGGGCCTGCAGAACACATTAACTTTGCGCTTGTAGAAATGACAGTCTTGCAATTACC
macaca_fascicularis  ACAACATGGTTCCATGATTTTCCTGTGGCACACAATGGAACGGGCTTGCAAAACACATTAATTTTGCACTTGTAGAAATGACAGTCTTGCAATTACC
                     ************************* ********** *** **** **** ********** ***** *****************************

homo_sapiens         ATTCATTGTGCTTGATGCTAATTTAATGCAGTCATTTCCTTGCCAAAGTAATGTCTGAATGCAATCAAGTAAGCACTAATGCCTGCGAGATTCCACT
pan_troglodytes      ATTCATTGTGCTTGATGCGAATTTAATGCAGTCGTTTCCTTGCCAAAGTAATGTCTGAATGCAATCAAGTAAGCACTAATGCCTGCAAGATTCCACT
gorilla_gorilla      ATTCATTGTGCTTGATGCTAATTTAATGCAGTCATTTCCTTGCCAAAGTAATGTCTGAATGCAATCAAGTAAGCACTAATGCCTGTGAGATTCCACT
macaca_fascicularis  ATTCATTGTGCTTGATGCTAATTTAATGCAGTCATTTCCTTGCCAAAGTAATGTCTGAATGCAATCAAGTAAGCACTAATGCCTGAGAGATTCCAGT
                     ****************** ************** ***************************************************  ******** *

homo_sapiens         ACATAAGTACCAGTTTAAAGAAAATC
pan_troglodytes      ACATAAGTACCAATTTAAAGAAAATC
gorilla_gorilla      ACATAAGTACCAATTTAAAGAAAATC
macaca_fascicularis  ATGTAAGTACCAATTTAAAGAAAATC
                     *  ********* *************
```

**Supplementary Figure 7. Multiple species alignment of HACNS_954 (510 bp HAR located within intron 18 of *CNTNAP2*)**

Human Accelerated Conserved Non-coding Sequence 954 (HACNS_954) contains six nucleotides that are fixed in all humans but are absent in other primates (nucleotides highlighted in red and blue, respectively). *H. sapiens* = human; *P. troglodytes* = chimpanzee, *G. gorilla* = western gorilla; *M. fascicularis* = crab-eating macaque.

**Supplementary File 1. Figure 2 works cited.**


1. Smogavec M, Cleall A, Hoyer J, Lederer D, Nassogne MC, Palmer EE, et al. Eight further individuals with intellectual disability and epilepsy carrying bi-allelic CNTNAP2 aberrations allow delineation of the mutational and phenotypic spectrum. J Med Genet. 2016;53(12):820-7.

2. Freri E, Castellotti B, Canafoglia L, Ragona F, Solazzi R, Vannicola C, et al. Severe epilepsy in CNTNAP2-related Pitt-Hopkins-like syndrome successfully treated with stiripentol. Seizure. 2021;88:143-5.

3. Zweier C, de Jong EK, Zweier M, Orrico A, Ousager LB, Collins AL, et al. CNTNAP2 and NRXN1 are mutated in autosomal-recessive Pitt-Hopkins-like mental retardation and determine the level of a common synaptic protein in Drosophila. Am J Hum Genet. 2009;85(5):655-66.

4. Mittal R, Kumar A, Ladda R, Mainali G, Aliu E. Pitt Hopkins-Like Syndrome 1 with Novel CNTNAP2 Mutation in Siblings. Child Neurol Open. 8: © The Author(s) 2021.; 2021. p. 2329048x211055330.

5. Watson CM, Crinnion LA, Tzika A, Mills A, Coates A, Pendlebury M, et al. Diagnostic whole genome sequencing and split-read mapping for nucleotide resolution breakpoint identification in CNTNAP2 deficiency syndrome. Am J Med Genet A. 2014;164a(10):2649-55.

6. Rodenas-Cuadrado P, Pietrafusa N, Francavilla T, La Neve A, Striano P, Vernes SC. Characterisation of CASPR2 deficiency disorder--a syndrome involving autism, epilepsy and language impairment. BMC Med Genet. 2016;17:8.

7. Karaca E, Harel T, Pehlivan D, Jhangiani SN, Gambin T, Coban Akdemir Z, et al. Genes that Affect Brain Structure and Function Identified by Rare Variant Analyses of Mendelian Neurologic Disease. Neuron. 2015;88(3):499-513.

8. Riccardi F, Urquhart J, McCullagh G, Lawrence P, Douzgou S. A patient with a novel CNTNAP2 homozygous variant: further delineation of the CASPR2 deficiency syndrome and review of the literature. Clin Dysmorphol. 2019;28(2):66-70.

9. Parrini E, Marini C, Mei D, Galuppi A, Cellini E, Pucatti D, et al. Diagnostic Targeted Resequencing in 349 Patients with Drug-Resistant Pediatric Epilepsies Identifies Causative Mutations in 30 Different Genes. Hum Mutat. 2017;38(2):216-25.

10. Strauss KA, Puffenberger EG, Huentelman MJ, Gottlieb S, Dobrin SE, Parod JM, et al. Recessive symptomatic focal epilepsy and mutant contactin-associated protein-like 2. N Engl J Med. 2006;354(13):1370-7.

11. Jackman C, Horn ND, Molleston JP, Sokol DK. Gene associated with seizures, autism, and hepatomegaly in an Amish girl. Pediatr Neurol. 2009;40(4):310-3.

**Supplementary File 2. Figure 3 works cited.**


1. Petrin AL, Giacheti CM, Maximino LP, Abramides DV, Zanchetta S, Rossi NF, et al. Identification of a microdeletion at the 7q33-q35 disrupting the CNTNAP2 gene in a Brazilian stuttering case. Am J Med Genet A. 2010;152a(12):3164-72.

2. Nord AS, Roeb W, Dickel DE, Walsh T, Kusenda M, O'Connor KL, et al. Reduced transcript expression of genes affected by inherited and de novo CNVs in autism. Eur J Hum Genet. 2011;19(6):727-31.

3. Egger G, Roetzer KM, Noor A, Lionel AC, Mahmood H, Schwarzbraun T, et al. Identification of risk genes for autism spectrum disorder through copy number variation analysis in Austrian families. Neurogenetics. 2014;15(2):117-27.

4. Lesca G, Rudolf G, Labalme A, Hirsch E, Arzimanoglou A, Genton P, et al. Epileptic encephalopathies of the Landau-Kleffner and continuous spike and waves during slow-wave sleep types: genomic dissection makes the link with autism. Epilepsia. 2012;53(9):1526-38.

5. Toma C, Pierce KD, Shaw AD, Heath A, Mitchell PB, Schofield PR, et al. Comprehensive cross-disorder analyses of CNTNAP2 suggest it is unlikely to be a primary risk gene for psychiatric disorders. PLoS Genet. 2018;14(12):e1007535.

6. Gregor A, Albrecht B, Bader I, Bijlsma EK, Ekici AB, Engels H, et al. Expanding the clinical spectrum associated with defects in CNTNAP2 and NRXN1. BMC Med Genet. 2011;12:106.

7. Girirajan S, Dennis MY, Baker C, Malig M, Coe BP, Campbell CD, et al. Refinement and discovery of new hotspots of copy-number variation associated with autism spectrum disorder. Am J Hum Genet. 2013;92(2):221-37.

8. Prasad A, Merico D, Thiruvahindrapuram B, Wei J, Lionel AC, Sato D, et al. A discovery resource of rare copy number variations in individuals with autism spectrum disorder. G3 (Bethesda). 2012;2(12):1665-85.

9. Mikhail FM, Lose EJ, Robin NH, Descartes MD, Rutledge KD, Rutledge SL, et al. Clinically relevant single gene or intragenic deletions encompassing critical neurodevelopmental genes in patients with developmental delay, mental retardation, and/or autism spectrum disorders. Am J Med Genet A. 2011;155a(10):2386-96.

10. Pippucci T, Licchetta L, Baldassari S, Palombo F, Menghi V, D'Aurizio R, et al. Epilepsy with auditory features: A heterogeneous clinico-molecular disease. Neurol Genet. 2015;1(1):e5.

11. Veerappa AM, Saldanha M, Padakannaya P, Ramachandra NB. Family-based genome-wide copy number scan identifies five new genes of dyslexia involved in dendritic spinal plasticity. J Hum Genet. 2013;58(8):539-47.

12. Mefford HC, Muhle H, Ostertag P, von Spiczak S, Buysse K, Baker C, et al. Genome-wide copy number variation in epilepsy: novel susceptibility loci in idiopathic generalized and focal epilepsies. PLoS Genet. 2010;6(5):e1000962.

13. Elia J, Gai X, Xie HM, Perin JC, Geiger E, Glessner JT, et al. Rare structural variants found in attention-deficit hyperactivity disorder are preferentially associated with neurodevelopmental genes. Mol Psychiatry. 2010;15(6):637-46.

14. Friedman JI, Vrijenhoek T, Markx S, Janssen IM, van der Vliet WA, Faas BH, et al. CNTNAP2 gene dosage variation is associated with schizophrenia and epilepsy. Mol Psychiatry. 2008;13(3):261-6.

15. Al-Murrani A, Ashton F, Aftimos S, George AM, Love DR. Amino-Terminal Microdeletion within the CNTNAP2 Gene Associated with Variable Expressivity of Speech Delay. Case Rep Genet. 2012;2012:172408.

16. Laffin JJ, Raca G, Jackson CA, Strand EA, Jakielski KJ, Shriberg LD. Novel candidate genes and regions for childhood apraxia of speech identified by array comparative genomic hybridization. Genet Med. 2012;14(11):928-36.

17. Lee IS, Carvalho CM, Douvaras P, Ho SM, Hartley BJ, Zuccherato LW, et al. Characterization of molecular and cellular phenotypes associated with a heterozygous CNTNAP2 deletion using patient-derived hiPSC neural cells. NPJ Schizophr. 2015;1.

18. Eriksson MA, Lieden A, Westerlund J, Bremer A, Wincent J, Sahlin E, et al. Rare copy number variants are common in young children with autism spectrum disorder. Acta Paediatr. 2015;104(6):610-8.

19. Centanni TM, Sanmann JN, Green JR, Iuzzini-Seigel J, Bartlett C, Sanger WG, et al. The role of candidate-gene CNTNAP2 in childhood apraxia of speech and specific language impairment. Am J Med Genet B Neuropsychiatr Genet. 2015;168(7):536-43.

20. Bakkaloglu B, O'Roak BJ, Louvi A, Gupta AR, Abelson JF, Morgan TM, et al. Molecular cytogenetic analysis and resequencing of contactin associated protein-like 2 in autism spectrum disorders. Am J Hum Genet. 2008;82(1):165-73.

21. Koshimizu E, Miyatake S, Okamoto N, Nakashima M, Tsurusaki Y, Miyake N, et al. Performance comparison of bench-top next generation sequencers using microdroplet PCR-based enrichment for targeted sequencing in patients with autism spectrum disorder. PLoS One. 2013;8(9):e74167.

22. Chiocchetti AG, Kopp M, Waltes R, Haslinger D, Duketis E, Jarczok TA, et al. Variants of the CNTNAP2 5' promoter as risk factors for autism spectrum disorders: a genetic and functional approach. Mol Psychiatry. 2015;20(7):839-49.

23. Chen XS, Reader RH, Hoischen A, Veltman JA, Simpson NH, Francks C, et al. Next-generation DNA sequencing identifies novel gene variants and pathways involved in specific language impairment. Sci Rep. 2017;7:46105.

24. Zhou WZ, Zhang J, Li Z, Lin X, Li J, Wang S, et al. Targeted resequencing of 358 candidate genes for autism spectrum disorder in a Chinese cohort reveals diagnostic potential and genotype-phenotype correlations. Hum Mutat. 2019;40(6):801-15.
